# Alfred Russel Wallace’s Darwinian Opposition to Eugenics

**DOI:** 10.1007/s10739-024-09792-6

**Published:** 2024-11-15

**Authors:** David Stack

**Affiliations:** Department of History, University of Reading, Whiteknights, P. O. Box 218, Reading, RG6 6AA UK

**Keywords:** Alfred Russel Wallace, Eugenics, Galton, Darwinism, Natural selection

## Abstract

This article revisits the question of Alfred Russel Wallace’s relationship to eugenics and explores the basis of Wallace’s consistent rejection of attempts to label him a eugenicist. Whereas some scholars have identified an ‘ambiguity’ or ‘tension’ between Wallace’s hereditarianism and his libertarianism and maintained – despite Wallace’s statements to the contrary – that he was, in some senses, a eugenicist, this article argues that Wallace’s oft-repeated claims he was not a eugenicist are fully justified. By exploring Wallace’s relationship with Francis Galton using a hitherto neglected correspondence between the two concerning the establishment of a proposed laboratory, and Wallace’s criticism of non-Darwinian evolutionary mechanisms in the writings of William Bateson and others, this article situates Wallace’s opposition to eugenics in his broader ultra-Darwinian agenda. The article concludes by arguing that it is misleading to characterise Wallace as a eugenicist, and that doing so tends to obscure and confuse our understanding of his thought.

## Introduction

In January 1913 the American feminist and suffrage campaigner Wenona Marlin wrote to Alfred Russel Wallace asking him to use his “high position” and “authority” to “stir up a widespread interest among Eugenists both in England and the United States on the need to “improve the race.”^1^ Marlin’s appeal was prompted by an interview Wallace had given to the London-based *Daily News & Leader*, reprinted in the *New York Times*, in which he had argued that human intellect and morals were largely unchanged from the time of ancient Egypt and that “the average of mankind will remain the same until natural selection steps in to raise it” (Wallace 1913b). From this, Marlin, herself a eugenicist alarmed by immigration, prison statistics, and the tax burden, had assumed that Wallace shared her views. We do not have Wallace’s reply, but we can infer what he might have said from another interview he had given a few months earlier to the *Millgate Monthly* magazine. Speaking with an “‘energy” that surprised his interviewer, the eighty-nine-year-old Wallace had objected that “you must not dream that I approve of any of the modern eugenic heresies.” He was, he explained, “a little sore on this point” because a popular scientific publication had described him as “spending the evening of my days in furthering the teaching of eugenics. Wherever did I advocate any such preposterous theories?” (Rockell 1912).

The early 20th century association of Wallace with eugenics might be explained away as a by-product of attempts by contemporary eugenicists – led first by Darwin’s half-cousin Francis Galton and then by his fourth son, Leonard Darwin – to actively claim Darwinism as their inspiration (Berra 2019). But the fact that the association, which so angered Wallace, continues in the early 21st century, suggests that context was not the only driver. It is also the case, as Diane B. Paul has pointed out, that the recurrent connection feeds on certain seeming “ambiguities in Wallace’s thought” (Paul 2008, p. 274). By this she meant the apparent tension between, on the one hand, Wallace’s acceptance of the “‘ostensible problem that eugenics addressed – the need to improve the hereditary quality of the race” by selective breeding – and, on the other, his “general and fundamental objection” to all schemes of legislative enactment and “interference with personal freedom” (Paul 2008, p. 274). Wallace was both a hard hereditarian, who argued that “no definite advance in morals can occur in any race *unless there is some selection or segregative agency at work”* (Wallace 1913a, p.46), and a committed libertarian who opposed all statist interventions to control reproduction. From this dual characterization it is easy to conclude that Wallace occupied an intellectually conflicted, and perhaps even contradictory, position. How, Paul wondered, could Wallace be a hereditarian and a libertarian? In particular, she pondered, how could the enthusiasm of Wallace’s “never repudiated” 1870 review of Galton’s *Hereditary Genius* (Wallace 1870; Paul 2008, p. 264) be reconciled with the vehemence of his 1912 condemnation of eugenics? Fichman (2019), building on Paul’s analysis, maintained that not only was Wallace’s position on eugenics “complicated” and “more complex than is usually allowed” (p. 206, p. 214), but also, despite his protestations to the contrary, that Wallace advocated “a certain form of eugenics” (p. 214), which Fichman called “a form of ‘voluntary eugenics’” (p. 215). More recently, James T. Costa and George Beccaloni (2023) have argued that Wallace came to embrace “a passive eugenical means to improve society” (p. 27).

It is noteworthy to find so many historians – and historians sympathetic to Wallace at that – so clearly contradicting Wallace’s assessment of his own position. Their argument, which boils down to an assertion that Wallace was a *partial* eugenicist, itself rests in part on a supposed distinction between *positive* eugenics – attempts to improve a population through a disproportionate breeding of those deemed desirable – and *negative* eugenics – attempts to eliminate the undesirable elements in a population group through restrictions on reproduction, including segregation and sterilization. Wallace, it is implied, accepted positive eugenics even as he rejected its more obviously anti-libertarian, and morally questionable, negative variant. It is a neat argument, but one we should reject, for several reasons. First, although sometimes deployed by eugenicists in the latter years of Wallace’s life (e.g. Schuster 1912, p. 235), the positive / negative eugenics dichotomy is largely anachronistic. These were not terms used by Galton in any of his major writings and their use only really took off, initially in the USA, at the end of the First World War (Popenone 1918, p. 162; McLaughlin 1919, pp. 53–54). It was not, that is, a distinction that would have had much meaning for, or been familiar to, Wallace. Second, although useful in understanding different strands of eugenic thought, historians of eugenics have long been aware that the “positive” and “negative” labels are not stable, discrete or exclusive, terms (Stepan 1991) and we are only liable to delude ourselves if we assume that imposing such a distinction explains anything about Wallace. Because third, when Wallace tells us that he rejects eugenics he does so on grounds that are equally applicable to positive and negative variants. When Wallace characterized eugenics, in his interview with the *Millgate Monthly*, as “the meddlesome interference of an arrogant, scientific priestcraft” (Rockell 1912, p. 663), it was a complaint that might have been made both against attempts to encourage certain types of breeding (positive eugenics) and measures to discourage other types of breeding (negative eugenics). For Wallace, both variants rested on the “arrogant” assumption that a select few (the “scientific priesthood”) could replace or improve upon natural selection.

Rather than attempting to reconcile Wallace to any form of eugenics, this article will seek to demonstrate, through a detailed exploration of Wallace’s relationship with Galton, that Wallace’s oft-repeated claims that he was not a eugenicist were fully justified. More than this, we will argue, that the idea of “ambiguity and ideological tension” in Wallace’s thought – long a staple of Wallace scholarship (Durant 1979, p. 45) – is misleading when it comes to understanding his attitude to eugenics. It was an attempt to iron-out supposed ambiguities that led Paul to consider the possibility that Wallace prioritized his “broader socio-political commitments,” especially his egalitarianism, anti-statism and views on women and marriage, over his hereditarianism (Paul 2008, p. 264). Paul, it should be stressed, did not make a simplistic argument about non-scientific factors trumping scientific ones: her case was self-consciously presented as a starting point rather than a conclusion, and acknowledged that Wallace rejected Galton’s eugenics on grounds of practicality as much as principle (Paul 2008, p. 276).^2^ Nonetheless, it is fair to say that the weight of her argument, especially as extended by Fichman (2019, p. 208), tended to emphasize the apparent tension between Wallace’s science and his extra-scientific concerns. This article takes Paul’s analysis in a different direction.

Beginning from Wallace’s claim that his opposition to eugenics was *predicated* upon his Darwinism, we seek to emphasize the consistency rather than the tension in Wallace’s anti-eugenics stance. “The world,” as Wallace put it in 1912, “does not want the eugenist to set it straight” precisely because natural selection could resolve any problems if only it were allowed to operate fully and freely (Rockell 1912, p. 663; Wallace 1893). Without falling into the trap of investing contested concepts such as “science” and “non-science” with more meaning than they deserve, it is striking that Wallace repeatedly framed his opposition to eugenics in terms of his understanding of evolutionary science; exploring why he did this, and what he meant by it, fits well with other recent developments in Wallace scholarship. First, as Weber (2010) has shown in relation to Wallace’s position on vaccination, rather than assuming that liberty trumped science in Wallace’s thinking, it is more illuminating to seek to understand and emphasize the scientific basis of Wallace’s libertarianism. Second, the core of our case – that Wallace’s opposition to eugenics was rooted in an understanding of Darwinism as the “elimination of the unfit” – builds upon and reinforces Charles H. Smith’s arguments about continuity in Wallace’s thought (Smith 2004), and Wallace’s consistent rejection of any parallel between a *predetermined* artificial selection on the one hand and *adaptation*, as “a function of environmental engagement, in natural selection,” on the other (Smith 2012a, p. 203). Third, our characterization of Wallace’s disillusionment with Galton forming part of a broader defence of Darwinism during the period of the so-called eclipse of Darwinism (Bowler 1983, 1988) is consistent with Costa and Beccaloni’s (2023) recent uncovering of the outline plan for Wallace’s final, unpublished, book which was envisaged as a defence of Darwinism against mutationism and Mendelism.

To understand why Wallace rejected eugenics we will begin by exploring what it was that Wallace admired in Galton’s writings, and then chart how this admiration fractured on two key questions.

## Wallace and Galton

A plethora of predisposing factors might have led Wallace to endorse eugenics. High among these was the personal regard in which he held Galton. The two men had first met in the mid-1860s, with Wallace describing Galton as among the “scientific friends” with whom he was “most intimate” when living in London between 1862 and 1870 (Wallace 1905, p. 34). The basis of this intimacy was unlikely to have been social (they came from starkly contrasting backgrounds) or political (Wallace’s inclinations were radical, Galton’s conservative), and was almost certainly primarily, broadly speaking, scientific. In a host of ways their intellectual interests and experiences overlapped and intersected. Both had travelled extensively (Galton in Africa, Wallace in South America and Asia) and were pioneers in biogeography and population level studies. Both too had derived an early understanding of human nature from phrenology, and, in the mid-1860s, were leading the way in extending the Darwin-Wallace mechanism of natural selection into the understanding of the human condition. Wallace’s 1864 paper, “The Origin of Human Races,” cleared the path for Galton’s 1865*MacMillan’s Magazine* articles on “Hereditary Character and Talent,” and raised the specter of the frustration of natural selection in civilized societies, that was to loom so large in Galton’s eugenic thinking.

Towards the end of 1868 Wallace accepted Galton’s offer of work as an Examiner, made on behalf of the Council of the Royal Geographical Society, and the next year he was an early and enthusiastic reader of *Hereditary Genius*.^3^ His subsequent 1870 review, in the first volume of *Nature*, is noteworthy for the warmth of its endorsement: *Hereditary Genius*, Wallace concluded, marked Galton out “as an original thinker” whose “book will take rank as an important and valuable addition to the science of human nature’ (Wallace, 1870, p. 503).^4^ This was not the standard response. Initial reactions were “generally tepid and sometimes hostile” (Gilham 2001a, pp. 171–172), and it was only later, with the 1892 reissue, that Galton’s first book-length foray into the territory of what would become eugenics, won an enthusiastic readership (Gokygit 1994). Yet for Wallace, the book’s concluding chapters contained “some of the most startling and suggestive ideas to be found in any modern work” (Wallace 1870, p. 502).

In the 1870s and 1880s the two men remained on friendly terms. In 1874, for example, we find Wallace writing to Galton to recommend the folklorist and entomologist W. F. Kirby (1844–1912) as a librarian for the Geographical Society, and in 1886, in preparation for his upcoming US lecture tour, Wallace asked Galton for the loan of some prepared lantern slides illustrating the “*progressive accuracy* of the laws of deviation.”^5^ In 1891 we find them asking after each other’s spouses, and Wallace invited Galton to visit him and Annie in Broadstone.^6^ Thereafter, however, the intimacy seems to have cooled. In 1904 it was a distinctly frosty sounding Wallace who told “Mr. Galton” that he had not returned “your ‘form’,” asking about his ancestors, because he knew “nothing special” of them and was, in any case, engaged in writing his autobiography, and could “only say generally that *none* of my known relatives are or were ‘noteworthy’.”^7^ It does not require a great empathetic leap to detect an edge in the response of the (relatively) socially disadvantaged Wallace, and Flannery (2011 p. 96) was correct to detect a similar antagonism in Wallace’s speech at the Linnean Society’s 50th anniversary of the joint-reading of the Darwin-Wallace papers (Anon. 1908, pp. 5–11). But although any sense of personal warmth seems to have dissipated in their dotage, there was never an outright clash between the two men.^8^

Spiritualism and religion, which one might have anticipated as a potential area of disagreement, never seemed to trouble their relationship, and only surfaced in their private correspondence on two occasions. On the first, in 1874, Galton initiated a conversation, asking, ostensibly on behalf of a friend, about the “best mediums” to contact in London.^9^ Ten years later, Wallace wrote to suggest that Galton’s discussion of the “objective efficacy of prayer” in *Inquiries into Human Faculty* (1883) – the book which coined the term eugenics – was misleading, and to propose that Galton read the discussion of the impact of specific prayers in Wallace’s *Miracles and Modern Spiritualism* (1875).^10^ This latter exchange is particularly interesting because it seemingly centered not on a question of belief per se, but upon scientific methodology. “I really hope you will go into this as a mere question of scientific fact,” Wallace cautioned, without any acknowledgement of the notoriously malleable boundaries of science and spiritualism (Noakes 2012)^11^ Wallace, for his part, was relaxed about Galton’s anti-religious sniping – “I do not hold any Christian doctrines whatever,” Wallace told an interviewer in 1898 – and in his published writings sometimes actively revelled in Galton’s attacks on the church as an institution (Anon. 1898).^12^

What tied Wallace and Galton together in the 1870s and 1880s was their shared championing of hard hereditarianism, at a time when that position was unfashionable. In his very first writings on human evolution in 1865 Galton had declared it “an approximately correct view of the origin of our life, if we consider our own embryos to have sprung immediately from those embryos whence our parents were developed, and these from the embryos of *their* parents, and so on for ever.” There were, said Galton, “but few instances in which habit even seems to be inherited” (Galton 1865, p. 322). At this point, however, Wallace was more open to a “‘softer” understanding. In 1868, reading Darwin’s *The Variation of Animals and Plants under Domestication* (1868), Wallace expressed enthusiasm for Darwin’s “ingenious” theory of pangenesis, writing to Charles Lyell to tell that:

The hypothesis is *sublime* in its simplicity and the wonderful manner in which it explains the most mysterious of the phenomena of life. To me it is *satisfying* in the extreme. I feel I can never give it up, unless it be *positively* disproved, which is impossible, or replaced by one which better explains the facts, which is highly improbable. (Wallace 1905, pp. 221–222)

Wallace’s ardor was hardly unique – even Galton expressed some limited admiration for pangenesis in the first edition of *Hereditary Genius –* but it soon cooled as he read, in the *Proceedings of the Royal Society* and the pages of *Nature*, about the results of Galton’s attempts to test the hypothesis. The results of Galton’s blood transfusion experiments on rabbits, “staggered” Wallace, almost as much as they irritated Darwin. In his autobiography, Wallace recalled that the experiments had provided “the very disproof I had thought impossible,” and thereafter he considered Galton’s experiments a significant landmark in disproving Lamarckian theories of acquired characters (Wallace 1900, pp. 315–316).

Not only, that is, were Wallace and Galton aligned in their understanding of inheritance, it was Galton who led Wallace on this point. According to Charles H. Smith’s intriguing attempt to enumerate the most important people in Wallace’s intellectual life by creating a weighted index of citations, Galton was one of Wallace’s ten most cited authorities.^13^ We can get a flavor of what this means by examining Wallace’s most complete statement of his science, *Darwinism: an Exposition of the Theory of Natural Selection with Some of its Applications* (1889).^14^ Some of the citations are relatively neutral, usually examples from Galton’s travel writings, but many explicitly endorse Galton’s view of inheritance and link it to August Weismann’s notion of the “germ-plasm” (Wallace 1889a, p. 220fn, p. 464). There were, Wallace acknowledged, differences between the anthropologist Galton and the biologist Weismann; nonetheless, he claimed, they should be “associated as discoverers of what may be considered (if finally established) the most important contribution to the evolution theory since the appearance of the *Origin of Species*” (Wallace 1889a, b, p. 442fn, p. 414). Ultimately it was Weismann who Wallace credited with having led him to feel “compelled to discard Darwin’s view,” and the German was cited even more frequently than Galton in *Darwinism*. But admiration for Weismann only enhanced Wallace’s regard for Galton and linking the two men’s names became a recurrent refrain.^15^ In 1889 he even drew the connection to the attention of Weismann’s English translator, Edward Bagnal Poulton, arguing that Galton had shown “a remarkable anticipation of Weismann’s theories, which I think should be noticed in a preface to the translation of his book."^16^  

For Wallace, the work of Weismann and Galton was complementary, and mutually reinforcing, and Galton deserved acknowledgment for leading the way. Wallace had been particularly impressed by Galton’s “A Theory of Heredity” (Galton 1876), which was read before the Anthropological Institute, and then reprinted in a revised form in the Institute’s journal in 1876. Upon reading it, Wallace wrote to tell Galton “how immensely I was please[d] & interested with your last paper in the Anthrop Journal. Your ‘Theory of Heredity’ seems to me most ingenious & a decided improvement on Darwin’s It gets over some of the great difficulties & the enormous controversies of his Pangenesis.”^17^ Ten years later, in 1886, we again find him writing to Galton, in similarly effusive terms, to express how he was “delighted with your address at the Brit. Ass. on *Heredity of Stature.*”^18^ What so pleased Wallace about these two papers was the same quality that later pleased him when he read Weismann’s *The Germ Plasm* (1893): they offered Wallace confirmation of the strength of natural selection and the non-heritability of acquired characters.^19^ Writing to Poulton in 1888, Wallace claimed that Galton’s “Regression towards Mediocrity” and Weismann’s “Panmixia” taken together constituted a full answer to Herbert Spencer and the notion of the inheritance of acquired characters.^20^

Wallace’s admiration for Galton on this point never wavered. His 1890 judgment that Galton had “studied the whole subject of human faculty in the most thorough manner, and has perhaps thrown more light upon it than any other writer,” was never renounced (Wallace 1890, p. 327). Wallace’s consistent commitment to Galton’s insights of the 1870s and 1880s is the basis for what Paul calls the “ambiguities” in Wallace’s later thought; the implication being that they ought – logically if not morally – to have led him to a wholesale endorsement of Galton’s eugenics. But there is, of course, an alternative explanation. Rather than assuming Wallace diverged, or displayed an inconsistency, that requires explanation, what if it was Galton? To Wallace’s mind at least, in the 1890s it was Galton who wavered: first, in showing a lack of enthusiasm for establishing a laboratory to decisively disprove the Lamarckian and Neo-Lamarckian theories which they both rejected, and second, in succumbing to the siren song of organic stability, which Wallace regarded as antagonistic to natural selection.

### The Proposed Laboratory

In 1891 Wallace initiated a correspondence with Galton, the significance of which has not previously been fully explored. In a series of letters, Wallace pushed a reluctant Galton to take steps to institute some “combined and systematic effort to carry out experiments for the purpose of deciding the two great fundamental but disputed points in organic evolution:” the inheritance of acquired characters and the extent of hybrid sterility. A committee of either the British Association or Royal Society, Wallace argued, was needed for a sufficient sum to be raised for “an *Institute for experimental enquiry* into the *fundamental data* of *biology*.” 21 Wallace’s initial approach was prompted by an encounter with the entomologist Theodore Dru Alison Cockerell (1866–1948) – “a very acute and thoughtful young naturalist” according to Wallace – who had recently taken a post at the Natural History Museum in London. 22 A letter from Cockerell dated 2 February 1891 had, Wallace explained, “set me going.” The letter itself is unlocated, so we cannot be entirely certain of what it said, but as Wallace refers to the letter “giving Romanes’s reply” – and responds by sketching “a series of a dozen sets of experiments to test the two questions of ‘*heredity of acquired characters’* and the ‘amount of sterility in the hybrid between closely allied species,’ as well as a few to test the questions of instinct in nest-building, and the ‘homing’ power of dogs, cats, & c.” – we can confidently assume that it outlined George Romanes’s advocacy of the inheritance of acquired characters.^23^

Wallace’s urgency contrasts sharply with Galton’s cautious reply. The idea of a laboratory, farm, or institute at which it would be possible to test questions of heredity was, Galton said, one that he had often toyed with himself, and which he had even made some moves towards in discussions with Edwin Ray Lankester (1847–1929) and Romanes. But the idea had come up short against practical difficulties, such as agreeing to a set of acquired character experiments which would be acceptable to both sides and allowing for the effects of confinement on fecundity. Although he could envisage a farm that “would bear a similar relation to Heredity – both plant and animal – that the Kew Observatory does to experimenters in Physical Science,” and which would pay its way by acting as a repository for books, family genealogies, and family portraits, which people would pay to have preserved, Galton doubted the initiative could be got off the ground.^24^ This was not the sort of response ever likely to satisfy Wallace, who saw every problem as the first step towards a solution, and while Galton fretted over the detail of hybrid experiments, Wallace breezily declared that: “I do not myself see *much* difficult in carrying out any of these.”^25^ There followed a flurry of letters in which Galton raised practical objections and Wallace sought to assuage them.

Beyond differences of temperament, the correspondence is revealing on several levels. First, it points to the hitherto unacknowledged possibility that Wallace might have played an inadvertent role in the institutional development of eugenics. It is, at the very least, interesting to find Wallace urging Galton to make use of the Royal Society, and to establish a laboratory to investigate inheritance, three years before the Society created, under Galton’s chairmanship, a Committee for Conducting Statistical Inquiries in the Measurable Characteristics of Plants and Animals (this became known as the Evolution Committee (Plants and Animals) in 1897), and over a decade prior to the instigation of the Eugenics Laboratory at UCL in 1904.^26^ Second, the letters confirm the regard in which Wallace held Galton, and the basis of that regard. *“You* are the man to do it,” Wallace told Galton in relation to establishing a laboratory, “both as the original starter of the theory of non-inheritance of acquired variations, the only experimenter on pan-genesis, and the man who has done most in experiment and resulting theory on allied subjects.”^27^ Galton himself was less convinced: “before anything could be done, even before asking for its serious consideration, a few *carefully* & fully worked out proposals of experiment ought I think to be drawn up. I mean just as much would have to be done if the proposer handed them in to the Govt. Grant or other committee, for a grant of money.”^28^

The last comment highlights the fact that it was Galton, not Wallace, who better embodied the changing structures and methods of late 19th century science. Despite his relatively humble background, Wallace’s assumptions were rooted in the working practices of gentleman scientists of an earlier era. “Surely,” Wallace mused in 1891, when discussing the possibility of an experimental farm, “some wealthy lord may be found to give a small tenantless farm for such a purpose.”^29^ Five years later, in 1896, he was still complaining that it would be “a disgrace to the *wealthy* Fellows of the [Royal] Society,” of whom he thought there were “scores,” if one could not be found to fund a Biological Farm.^30^ Galton, by contrast, was at the center of the committee-based structures and the mathematical turn of 1890s science. This was a milieu in which Wallace could not feel comfortable. In 1893 he declined Galton’s invitation to join the Royal Society’s Evolution Committee, partly on the grounds that he was unlikely to attend; he was no more a committee man than Darwin, but also because although he declared himself “greatly interested” he was, he demurred, a “poor” mathematician. Indeed, he went one step further, questioning how useful mathematics was in understanding natural selection: “It seems to me (though it may be quite wrong) that the mathematical treatment of the subject does not bring out some of the most interesting points as regards evolution by natural selection. For instance, what may be called irregular deviations from the mean are I think of great importance for nat. select.”^31^ This is a doubly significant comment, because it points towards what Wallace considered one of the limitations of the statistical method, which Galton increasingly favored, in maintaining a strict Darwinian interpretation.^32^

## Organic Stability and Transilient Variation

By the 1890s the experimental approach, which Wallace admired in Galton, had largely been forsaken in his statistical pursuit of human measurement and data collection.^33^ This, of course, had always been an important strand in Galton’s research. Although his 1865 *MacMillan Magazine* articles had focussed primarily on mental and moral qualities, by the time he gathered the papers that made up his *Inquiries into Human Faculty and Its Development* (1883) his interest in measuring physical characteristics was clear, and this was confirmed by the success of his Anthropometric Laboratory at the International Health Exhibition in South Kensington in 1884.^34^ By 1890 this shift in focus had led Galton to collect fingerprints. One result of his early research on fingerprints, which he was keen to share with Wallace, was his belief that they were fully developed in the early months of foetal life and were “not correlated with any other characteristics.” He continued:

They are the same in the lowest idiots as in ordinary persons. (I took the impressions of some 80 of these, so idiotic that the mostly could not speak, or even stand, at the great Darenth Asylum, Dartford). They are the same in clodhoppers as in the upper classes, and *yet* they are as hereditary as other qualities, I think. 35

From this he concluded that, because they did not correlate with vigor, wits, or any other trait, neither sexual nor natural selection could explain them: “They just go their own gait, uninfluenced by anything that we can find or reasonably believe in, of a *naturally selective influence*, in the plain meaning of the phrase.”^36^ We do not have Wallace’s reply, but we can get a flavor of what he must have said, from an 1895 letter in which Wallace reminds Galton: “I told you at the time you published your finger print articles that I thought your deductions from them as to Nat. Selection & Species & c. *all wrong* & you will see I still hold that view & give my reasons.”^37^

Those reasons were set out in what Wallace himself described as “an important article,” published in two parts under the title “The Method of Organic Evolution” in the *Fortnightly Review* of 1895. The first part of the article focused on Wallace’s critique of William Bateson’s *Materials for the Study of Variation* (1894), and it was only in the second part that Galton came directly into his line of fire.^38^ This is important to appreciate, because it illustrates the fact that by the mid-1890s Wallace saw himself engaged in a battle to defend Darwin’s legacy against a host of evolutionary biologists, including Raphael Weldon (1860–1906), Romanes, and Bateson, in which Galton was allied with “youthful newcomers to the field” who were seeking “to make their mark by modifying some part of Darwin’s doctrine” (Harrison 1989, p. 242). In response, Wallace was driven by a desire to defend Darwinism. Thus, despite judging Bateson’s book “one of the *most pretentious* and most *worthless - ‘as a contribution to the study of the problem of Species’ -* I have ever met with,” he had spent “nearly a month” in autumn 1894, “wading through… & writing a criticism of it - & of Galton who backs him up with his idea of ‘organic stability.’” 39 His dedication to this unpleasant task was rooted in his desire to combat what he took to be a concerted attack on “Darwin’s theory” (Wallace 1895).

The central “misconception” underlying Bateson’s book, said Wallace, was the idea that there were “definite positions of organic stability” that existed “independent of utility and therefore of natural selection,” and that these positions were reached by a process of “*discontinuous variation.*” Darwin, said Wallace, had identified two classes of variation: individual differences, which were small and numerous; and “sports,” which were large and rare. But whereas Darwin had seen “sports” as “quite unimportant,” Bateson had relabelled them “discontinuous variation” and made them “the all-important, if not the exclusive, means by which the organic world has been modified.” This “backward step in the study of evolution,” said Wallace, was traceable to Galton’s 1889 book *Natural Inheritance*, which was then restated in Galton’s paper on fingerprints read at the Royal Society (Galton 1891), before being adopted by Bateson, and then endorsed by Galton in his 1894 paper in *Mind* (Wallace 1895, p. 435, p. 437, p. 212. p. 216).

The supposed success of Bateson’s discontinuous variations, Wallace noted, depended upon Galton’s theory of organic stability, but that notion was “absolutely unintelligible and powerless unless in strict subordination to natural selection” (Wallace 1895, p. 444). Although Galton claimed that without “transilient” (his preferred term for discontinuous) variation there would be a regression to mediocrity, this misunderstanding arose from a failure to properly appreciate the power and unremitting action of the universal struggle for existence (Wallace 1895, pp. 437–438). Galton’s “organic stability,” Wallace concluded, had no explanatory value because it had “no meaning except that of harmonious adaptation to the environment as tested and maintained by natural selection” (Wallace 1895, p. 440). Galton and Bateson had made a mistake because they had failed to keep in view “the tremendous severity of this irresistible and never-ceasing process of selection” (Wallace 1895, p. 445).^40^

Although in his autobiography *Memories of My Life* Galton stressed the epoch-making impact of Darwin’s *Origin* on his own thinking (Galton 1908, p. 287), this should not blind us to the extent to which his understanding of evolution, and thus the foundation of his eugenic thinking, diverged from Darwin. The pangenesis dispute is well known, but arguably more significant was the criticism of Darwin that Galton began to develop twenty years later in his book *Natural Inheritance* (1889), in which he championed the evolutionary potential of “sports.” In the *Origin* Darwin had not entirely ruled out the possibility that “single variations” might lie at the root of a specific evolutionary change, but the overwhelming weight of his argument emphasized the insistent action of small, incremental changes accumulated over long periods of time. In *Natural Inheritance* Galton pushed in the opposite direction: “That the steps *may* be small and that they *must* be small are very different views; it is only to the latter that I object” (Galton 1889, p. 32). There was, he argued, a potential stability in “sports,” which might circumvent the need for natural selection: “sometimes a sport may occur of such marked peculiarity and stability as to rank as a new type, capable of becoming the origin of a new race with very little assistance on the part of natural selection” (Galton 1889, p. 30, p. 28). Two years later, in his paper on patterns in thumb and finger at the Royal Society Galton identified a stability which, he argued, could not be explained by either natural or sexual selection. Instead, he maintained, there was a distribution of individual varieties around typical centres. This led to the conclusion that Natural Selection had no monopoly of influence in creating genera or maintaining purity (Galton 1891, p. 22). His apostasy was completed three years later. In an article entitled “Discontinuity in Evolution,” published in *Mind*, Galton brought these insights together, concluding: “Many, if not most breeds, have had their origin in sports” (Galton 1894, p. 365).

His views, Galton said, had left him in what he thought was a “minority of one” until it was “with the utmost pleasure that I read Mr. Bateson’s work bearing the happy phrase in its title of ‘discontinuous variation,’ and rich with many original remarks and not a few trenchant expressions” (Galton 1894, p. 369). Galton, we should be clear, was not simply allowing for the importance of “sports” or “discontinuous variation,” he was insistent upon the need for *transilience*. Without an abrupt movement from one position of organic stability to another, he argued, any mere bend or divergence would regress: “I am unable to conceive the possibility of evolutionary progress except by transiliences, for, if they were merely divergences, each subsequent generation would tend to regress backwards towards the typical centre, and the advance that had been made would be temporary and could not be maintained” (Galton 1894, p. 0.368).

For Wallace, by contrast, the idea that species could form without natural selection or possess “specific characters” that were non-adaptive’ was “impossible” and “unthinkable.” 41 All such discontinuity theories were false: “owing to the constancy, universality, and extreme severity of elimination through survival of the fittest, such large and abrupt variations, except through some extraordinary and almost impossible concurrence of favourable conditions, can never permanently maintain themselves” (Wallace 1905, p. 213). The root problem, he told Poulton, was that “[n]either [Bateson] nor Galton appear to have any adequate conception of what Natural Selection is, or how impossible it is to escape from it. They seem to think that given a *stable* variation & natural selection must hide its diminished head!”^42^ What lay behind their mistake, Wallace explained in a letter to the entomologist and chemist Raphael Meldola (1849–1915), was their lack of scientific training:

I do not think species-*describing* is of any special use to the philosophical generaliser, but I do think the *collecting*, *naming*, & *classifying*, some extensive group of organisms *is* of great use, is, in fact, almost essential to any thorough grasp of the whole subject of the Evolution of species through *variation* & nat selection – I had *described* nothing when I wrote my papers on variation & c. (except a few fishes & palms from the Amazon) but I had *collected* & *made out* species, very largely, & had seen, to some extent, how curiously useful & protective their forms and colours often were, & all this was of a great use to me. I think the errors (as I consider them) of Galton and Bateson, are to a great extent due to a want of such training.^43^

In rejecting the notion that variation by “sports” or “monstrosities” formed part of the method of evolution, Wallace felt sure that he was aligned with Darwin.^44^ This, after all, was not a new argument.

Darwin had addressed saltationism directly in the *Origin* with his repeated incantation of the aphorism “*Natura non facit saltum.”*^45^ Similarly well-rehearsed were the arguments that could be made against Galton’s contention that discontinuity was necessary to avoid any regression to mediocrity. This argument against Darwinism had first surfaced in an 1867 review of the *Origin* in which Fleeming Jenkin, an engineer by profession, had maintained that natural selection could not possibly work at the incremental rate Darwin suggested because, without a caesural break, any advantageous mutations would be swamped and diluted out of existence within a few generations (Fleeming Jenkin 1867; Hoquet 2024). Darwin had been troubled by the criticism and was commensurately grateful when Wallace addressed it in his review (Wallace 1867) of the Duke of Argyll’s *The Reign of Law* – a review which led Darwin to gush: “I must say I admire every word.”^46^

The essence of Wallace’s argument was that Jenkin had misunderstood how natural selection worked by confusing single variations – analogous to the artificial selection that a breeder might effect – with “the modifications which exist in nature.” This was the same point that Darwin expanded upon in a passage added to the fifth edition of the *Origin* in 1869, in which he made clear that he accepted that “single variations,” analogous to “when man selects” had only “a very poor chance of perpetuating its kind to the exclusion of the common form.” But what Jenkin failed to appreciate was that “certain variations, which no one would rank as mere individual differences, frequently recur owing to a similar organisation being similarly acted on,” and in such cases, if the variation were of beneficial nature, the original form would soon be supplanted by the modified form, through the survival of the fittest’ (Darwin 1869, pp. 104–105).^47^ Wallace, therefore, was confident in seeing his position as consistent with that of Darwin: “During the whole of Darwin’s life I can safely say that there was absolutely no difference whatever between Darwin’s views and my own on this subject.” This was a judgment that carried the implicit corollary that Galton’s understanding of evolution was not the same as Darwin’s.^48^

## Wallace, Galton, and Natural Selection

The significance of Galton’s divergence from Darwin for his estimation of the likely speed and success of eugenic reform needs to be acknowledged. His embrace of “discontinuous variation” was no small matter. It pointed not just to a speedier process, but also to fundamentally different assumptions about the evolutionary process that became central to eugenics and which Wallace did not share. First, the notion of organic stability and transilience – built upon a study of anthropometric measurements, which Wallace regarded as at best irrelevant – provided Galton with an understanding of human racial difference, which was independent of both natural selection and sexual selection (Galton 1894, p. 367). Second, whereas Wallace (and Darwin) regarded the evolution of races and species as “an enormously protracted process,” discontinuous variation opened up the possibility of much swifter change. This made eugenic advance, through well designed interventions, Galton maintained, much more plausible:

It does not seem to me by any means so certain as is commonly supposed by the scientific men of the present time, that our evolution from a brute ancestry was through a series of severally imperceptible advances. Neither does it seem by any means certain that humanity must linger for an extremely long time at or about its present unsatisfactory level. As a matter of fact, the Greek race of the classical times have surpassed in natural faculty all other races before or since, and some future race may be at least the equal of the Greek, while it is reasonable to hope that when the power of heredity and the importance of preserving valuable ‘transiliences’ shall have become generally recognized, effective efforts will be made to preserve them. (Galton 1894, p. 372)

Acknowledging the extent of this divergence from Darwinism by Galton weakens the Darwin-eugenics connection which the eugenicists worked so hard to cement and which historians have been too ready to accept. It also provides us with one possible solution to the supposed problem of ambiguities in Wallace’s attitude towards eugenics, by turning that problem on its head. Rather than inconsistency on Wallace’s part we might instead more reasonably focus on a change in Galton’s position. This, however, ought only to be part of our answer. Because if we go right back to Wallace’s “never repudiated” 1870 review of *Hereditary Genius* (Paul 2008, p. 264), we can see that even at that point he harbored significant doubts about Galton’s understanding of natural selection. Rather, that is, than presenting us with a problem in explaining Wallace’s later opposition to eugenics, the review represents a foreshadowing of the criticisms of Galton that Wallace was to express so strongly in the 1890s.

Our argument here turns on what Paul called Wallace’s “only one mild disagreement” (Paul 2008, p. 268) with Galton in the review, which related to Galton’s objection to Malthus’s urging of delayed marriage. Galton expressed the common degenerationist fear that it would be the most prudent – the middle classes – who would show restraint, and that the resultant differential reproduction would lead to racial decline. This reasoning was to become the basis for what Andre Pichot called “the great anthropological fable of the degeneration of the human race for lack of natural selection” (Pichot 2009, p. 120), which underpinned all future eugenic arguments. Wallace took issue with it from the first. Not because he disagreed about differential reproduction – which was a concern he and Darwin shared with Galton – but because, as he pointed out, evolution by natural selection is not a question of reproduction so much as of survival.^49^ His argument, that is, is that Galton failed to understand the power of natural selection: differential survival will cancel out any differential in the birth rate.

Not less striking is his exposition of the effects of prudential restraints on marriage, on the general character of a nation. If one class of people, as a rule, marry early, and another class marry late in life, the former have a double advantage, both in having on the average larger families, and in producing more generations in each century. But, by the supposition, it is the imprudent who gain this advantage over the prudent; and Mr. Galton therefore denounces the doctrine of Malthus, that marriage should be delayed till a family can be supported, unless the rule could be imposed on all alike. I hardly think that this argument is sound, and I doubt if the imprudent who make early marriages do, in the long run, increase more rapidly than the prudent who marry late. Increase of population depends less upon the number of children born, than on those which reach manhood; and I believe that the prudent man who has acquired some wealth and wisdom before he marries, will give to the world more healthy men and women, than the ignorant and imprudent youth, who marries a girl as ignorant and imprudent as himself. It is also to be remembered that the men who marry late often marry young wives, and have as good a chance of large families as the imprudent. (Wallace 1870, p. 503)

This may be judged only a “mild criticism” in the context of an otherwise overwhelmingly positive review, but it points to a fatal weakness in Galton’s understanding of natural selection. It confirms that there was always a *scientific* divergence between the two men, which we do not need to reach to politics or spiritualism to explain, and that Wallace stood with Darwin on this point. Darwin, having read Wallace’s piece, wrote to the co-founder of evolution by natural selection to declare “I was excessively pleased at your review of Galton, & I agree to every word of it.”^50^

## Wallace’s Darwinian Case against Eugenics

Twenty years later, starting with “Human Selection” (1890), which Wallace described as “the most important contribution I have made to the science of sociology and the course of human progress,” and continuing through to his final book, *Social Environment and Moral Progress* (1913), which appeared at a time when, as Wallace put it, “much is being written about checking degeneration and elevating the race to a higher level,” Wallace made a consistent argument against eugenics (Wallace 1913a, p. 141). In his own view, his later writings constituted “a complete refutation of all the superficial ideas as to the teaching of ‘natural selection’ applied to man, and also of the dangerous, because altogether unnatural, proposal to regulate the breeding of human beings by direct interference with individuals”(Wallace 1908a). The crux of his case, as he explained in his autobiography, was that “artificial elimination and selection, are both unscientific and unnecessary” (Wallace 1905, p. 209). By this he meant that any active eugenic interventions were both superfluous and premised upon a misunderstanding of how natural selection worked.

There was an undeniably political aspect to his arguments. Science and politics were not discrete endeavors, and Wallace frequently strayed into areas that we might consider extrinsic to science. Thus in “Human Selection, responding to Hiram Miner Stanley’s proposals (Stanley 1890) for “trained specialists” to control reproduction, Wallace made a moral argument (“nothing can be more objectionable”); a pragmatic argument (“such interference with personal freedom in matters so deeply affecting individual happiness will never be adopted by the majority of any nation”); and a libertarian one (“a general and fundamental objection”) to all schemes of legislative enactment (Wallace 1890, pp. 328–330). Similarly, in *Social Environment* he characterized eugenic legislation as “dangerous and detestable,” “totally unnecessary,” and “a much greater source of danger to morals and to the well-being of humanity than the mere temporary evils it seeks to cure. He also threw in a “slippery-slope” argument for good measure (“there is great danger in such a process of artificial selection by experts who would certainly soon adopt methods very different from those of the founder”), and highlighted the incompetence of the legislature (“a chance body of elected persons who are totally unfitted to deal with far less complex problems than this one, and as to which they are sure to bungle disastrously” (Wallace 1913a, pp. 127–129). But, for Wallace, none of these points were in conflict with or prior to his consistent contention concerning the power and sufficiency of natural selection.

Wallace shared the eugenicists’ concern that civilization possessed a tendency to frustrate the evolutionary process (Wallace 1864).^51^ He also shared their belief in biological inheritance as the long-term source of human progress and betterment. But unlike the eugenicists, Wallace acknowledged neither the need nor the efficacy of artificial selection. For one thing, Wallace judged the imperfections of civilization “mere temporary evils” when set beside the overwhelming power of natural selection which, even operating under inadequate social arrangements tended to produce an overbalance of good. He wrote:

Humanity - the essentially human emotion - has caused us to save the lives of the weak and suffering, of the pained or imperfect in mind or body. This has to some extent been antagonistic, to physical and even intellectual race-improvement; but it has improved us morally by the continuous development of the characteristic and owning grace of our human, as distinguished from our animal, nature. (Wallace 1890, pp.330–331)

This was similar to the argument that Darwin had made in the *Descent of Man* (1871) when he maintained that sympathy was itself an evolutionary inheritance and, on that basis, rejected any proto-eugenic actions that compromised “the noblest part of our nature” (Darwin 1871; vol. I, p. 168). Both men trusted in the power of natural selection, rather than succumbing to any eugenicist hyperbole about “race suicide.” But whereas Darwin was content to allow laissez-faire to do its work (Stack 2012), Wallace looked towards a fundamental societal reform that would unleash the unfettered power of a “truly natural selection:”

when we have cleansed the Augean stable of our existing social organisation, and have made such arrangements that all shall contribute their share of either physical or mental labour, and that all workers shall reap the full reward. Of their work, the future of the race will be assured by those laws of human developments that have led to the slow but continuous advance in the higher qualities of human nature. (Wallace 1890, pp. 330–331)

The eugenicists’ desideratum, the improvement of the race, Wallace concluded, would best be realized by a change in social organization that released the full power of natural selection. The form this argument took was framed by his politics but rooted in his science. Following his 1889 reading of Edward Bellamy’s utopian novel, *Looking Backward: 2000 − 1887* (1888), Wallace advocated for a socialist society in which women enjoyed economic independence, but justified this in terms of natural selection.^52^ As Wallace explained in an interview in the *Daily Chronicle* (Wallace 1893), the economic independence of women was desirable not only as a “social revolution” but also as a necessary step towards the full and effective operation of natural selection, which would obviate any argument for eugenics. The educated and emancipated women of the future, Wallace maintained, would reject coarse, sensual, idle, and selfish men, and thereby “cleanse society of the unfit,” without the need for artificial selection.^53^ Thus although his inspiration was a political utopia, Wallace’s reasoning was resolutely biological. It is this vision, which Wallace conceived as an explicit rebuttal to the eugenicists, that contemporaries and historians alike have mischaracterized as eugenic.

## Adaptation not Eugenics

To understand why this is a mischaracterization, we might usefully go back to the letter Wallace famously sent to Darwin in 1866, suggesting that the metaphor of natural selection had become a “stumbling block” to understanding and might be perspicaciously replaced with Spencer’s phrase “survival of the fittest,” as a more “plain expression of the facts.” Darwin’s term, Wallace complained, inadvertently implied active agency in a process that was nothing more or less than one of adaptation as a function of environmental engagement.^54^ This same understanding underlay Wallace’s argument that a more equal society would allow for “a continuous improvement of the race” (Wallace 1890, pp. 333–335). Wallace was not a late convert to sexual selection – a mechanism he regarded as “imaginary” – nor was he fully aligned with the “eugenical feminism” or “eugenization of love” arguments of New Woman authors such as Sarah Grand (Richardson 1999). His case was that a free and equal society was the necessary precondition for the full operation of natural selection, which would “come spontaneously into action” once women were freed from their economic subordination, and rendered a “powerful selective agency, able to reject the “idle and selfish,” the “diseased” and “weak in intellect” (Wallace 1913a, p. 132). A free and equal society, in which women enjoyed “a real choice in marriage,” that is, would “lead both to the more rapid elimination of the lower, and more rapid increase of the higher types of humanity” (Wallace 1905, pp. 209–210). There was no need for eugenics because in a fairer society “the deeper problem of the improvement of the race” (Wallace 1890, p. 337) would be addressed naturally by a process of evolutionary adaptation.

It is easy to see why contemporaries and historians alike have been tempted to think of this as a variant of eugenics. But the oddity and unfairness of labelling Wallace a eugenicist on the basis of writings which he explicitly conceived of as a statement of his *opposition* to eugenics, is also striking. What, we might ask, is gained by continuing to refer to Wallace with a label he repeatedly rejected? Even when the noun is moderated with a softening adverb such as “voluntary” or “passive” it still tends to obscure rather than illuminate our understanding. Eugenics is a notoriously nebulous term (Paul 2008, p. 272), but if it means anything, it means an active intervention in the evolutionary process. By contrast, at the heart of the “Wallacean view” (Smith 2012b, p. 9) of evolution sits a version of natural selection that leaves no space for predetermined ideals of fitness, only adaptation as “a function of environmental engagement” (Smith 2012a, p. 203). Certainly, Wallace was a hereditarian who believed that mental and moral traits were inherited and that a better biological future was both possible and desirable. But beyond a thin layer of confusion little is gained by labelling these views eugenics. What is lost, or at least obscured, is the distinctiveness of Wallace’s position. What this article hopes to have shown, by a detailed exploration of Wallace’s relationship with Galton, is that Wallace’s oft-repeated claims that he was not a eugenicist were fully justified.

## Footnotes

1 Wenona Marlin to Alfred Russel Wallace, 6 January 1913. “WCP551,” in Beccaloni, G. W. ed. *Ɛpsilon: The Alfred Russel Wallace Collection* accessed on 7 October 2024, https://epsilon.ac.uk/view/wallace/letters/WCP551 Hereafter all references are to the Wallace Correspondence Project (WCP) but are found in Epsilon < https://epsilon.ac.

2 Scholars have long acknowledged that there were not distinct states of “science” and “non-science,” and both “science” and the person of the “‘scientists” were constructions actively fashioned. See, for example, Ellis (2017).

3 Wallace to Galton, 15 December 1868. “WCP4664,” in Beccaloni, G. W. ed. *Ɛpsilon: The Alfred Russel Wallace Collection* accessed on 7 October 2024, https://epsilon.ac.uk/view/wallace/letters/WCP4664.

4 Darwin told Wallace he was “excessively pleased” with the review. To A.R. Wallace, 31 March 1870, Darwin Correspondence Project, “Letter no. 7154,” Darwin Correspondence Project (DCP). Hereafter all letters cited can be found here: https://www.darwinproject.ac.uk/.

5 Wallace to Galton, 15 January 1874. “WCP4145,” in Beccaloni, G. W. ed. *Ɛpsilon: The Alfred Russel Wallace Collection* accessed on 7 October 2024, https://epsilon.ac.uk/view/wallace/letters/WCP4145. Wallace to Galton 7 March 1886, “WCP4142,” in Beccaloni, G. W. ed. *Ɛpsilon: The Alfred Russel Wallace Collection* accessed on 7 October 2024, https://epsilon.ac.uk/view/wallace/letters/WCP4142.

6 Galton to Wallace, 5 February 1891. “WCP1515,” in Beccaloni, G. W. ed. *Ɛpsilon: The Alfred Russel Wallace Collection* accessed on 7 October 2024, https://epsilon.ac.uk/view/wallace/letters/WCP1515. Wallace to Galton 7 Feb. 1891. “WCP4137,” in Beccaloni, G. W. ed. *Ɛpsilon: The Alfred Russel Wallace Collection* accessed on 7 October 2024, https://epsilon.ac.uk/view/wallace/letters/WCP4137.

7 Wallace to Galton, 4 July 1904. “WCP4133,” in Beccaloni, G. W. ed. *Ɛpsilon: The Alfred Russel Wallace Collection* accessed on 7 October 2024, https://epsilon.ac.uk/view/wallace/letters/WCP4133.

8 There was a similar souring in Wallace’s attitude to Herbert Spencer, whose early writings he admired greatly but whose later writings, especially on the question of land nationalization, he regarded as “illogical” and “inconsistent.” See Wallace (1900; vol. 2, pp. 333–334).

9 Wallace suggested that he ask a Mr. Burns, of 15 Southampton Row: “He knows all the mediums & all about them.” Wallace also said that if Galton would provide “your friends’ name” he would send a card introducing a Mrs Guppy of Holloway. Wallace to Galton, 23 May 1874. “WCP4146,” in Beccaloni, G. W. ed. *Ɛpsilon: The Alfred Russel Wallace Collection* accessed on 7 October 2024, https://epsilon.ac.uk/view/wallace/letters/WCP4146.

10 On Wallace and prayer see Smith, Charles H. 2020. Alfred Russel Wallace Notes 13: Wallace on Prayer. https://people.wku.edu/charles.smith/essays/ARWNotes13%20Prayer.pdf).

11 Wallace to Galton, 27 July 1884. “WCP4143,” in Beccaloni, G. W. ed. *Ɛpsilon: The Alfred Russel Wallace Collection* accessed on 7 October 2024, https://epsilon.ac.uk/view/wallace/letters/WCP4143.

12 See also Wallace 1908c, 1913b); and Smith, Charles H., “Alfred Russel Wallace Notes 30. Wallace A Theist? Part I.” (2024). *Faculty/Staff Personal Papers.* Paper 346 [accessed 7 October 2024] https://digitalcommons.wku.edu/fac_staff_papers/346.

13 For more on Charles H. Smith’s work see: https://people.wku.edu/charles.smith/wallace/mostcite.htm.

14 In the text Galton is cited more often than Lyell, Lubbock, Lamarck, and Huxley; the number of mentions of Galton is only outstripped by those for Darwin, Spencer, Bates, and Weismann.

15 The biologists of Europe were, Wallace suggested in 1892, more accepting of Weismann and Galton than they had been of Darwin’s pangenesis (Wallace 1892). By 1908 he was certain that “all the chief biological thinkers and investigators” accepted Galton and Weismann’s view. i.e. it was not an exclusively eugenic belief (Wallace 1908c, p. 11).

16 Wallace to Edward Bagnal Poulton, 20 January 1889.“WCP4358,” in Beccaloni, G. W. ed. *Ɛpsilon: The Alfred Russel Wallace Collection* accessed on 7 October 2024, https://epsilon.ac.uk/view/wallace/letters/WCP4358.

17 Wallace to Galton, 3 March 1876. “WCP4140,” in Beccaloni, G. W. ed. *Ɛpsilon: The Alfred Russel Wallace Collection* accessed on 7 October 2024, https://epsilon.ac.uk/view/wallace/letters/WCP4140.

18 Wallace to Galton, 7 March 1886. “WCP4142,” in Beccaloni, G. W. ed. *Ɛpsilon: The Alfred Russel Wallace Collection* accessed on 7 October 2024, https://epsilon.ac.uk/view/wallace/letters/WCP4142.

19 Wallace to Raphael Meldola, 27 April 1893. “WCP4526,” in Beccaloni, G. W. ed. *Ɛpsilon: The Alfred Russel Wallace Collection* accessed on 7 October 2024, https://epsilon.ac.uk/view/wallace/letters/WCP4526 and Wallace to Meldola 10 June 1893. “WCP4527,” in Beccaloni, G. W. ed. *Ɛpsilon: The Alfred Russel Wallace Collection* accessed on 7 October 2024, https://epsilon.ac.uk/view/wallace/letters/WCP4527. On Meldola see Travis 2010.

20 Wallace to Poulton, 26 Septembr 1888. “WCP4353,” in Beccaloni, G. W. ed. *Ɛpsilon: The Alfred Russel Wallace Collection* accessed on 7 October 2024, https://epsilon.ac.uk/view/wallace/letters/WCP4353.

21 Wallace to Galton, 3 February 1891. “WCP1434,” in Beccaloni, G. W. ed. *Ɛpsilon: The Alfred Russel Wallace Collection* accessed on 7 October 2024, https://epsilon.ac.uk/view/wallace/letters/WCP1434.

22 It is unclear how the two men first met, but in November 1890 Wallace supported Cockerell’s attempts to secure a post at the Plymouth Biological Station. See Wallace to Theodore Dru Alison Cockerell, 2 November 1890. “WCP4210,” in Beccaloni, G. W. ed. *Ɛpsilon: The Alfred Russel Wallace Collection* accessed on 7 October 2024, https://epsilon.ac.uk/view/wallace/letters/WCP4210. See also Wallace to Cockerell 7 November 1890. “WCP4208,” in Beccaloni, G. W. ed. *Ɛpsilon: The Alfred Russel Wallace Collection* accessed on 7 October 2024, https://epsilon.ac.uk/view/wallace/letters/WCP4208.

23 Wallace to Cockerell 7 February 1891. “WCP4217,” in Beccaloni, G. W. ed. *Ɛpsilon: The Alfred Russel Wallace Collection* accessed on 7 October 2024, https://epsilon.ac.uk/view/wallace/letters/WCP4217. On Wallace and Romanes see Elsdon-Baker 2008.

24 Galton to Wallace, 5 February 1891. “WCP1515,” in Beccaloni, G. W. ed. *Ɛpsilon: The Alfred Russel Wallace Collection* accessed on 7 October 2024, https://epsilon.ac.uk/view/wallace/letters/WCP1515. “In all this,” he told Wallace, “I am keeping the Kew Observatory in view as a somewhat analogous institution.” Galton to Wallace 12 February 1891. “WCP2442,” in Beccaloni, G. W. ed. *Ɛpsilon: The Alfred Russel Wallace Collection* accessed on 7 October 2024, https://epsilon.ac.uk/view/wallace/letters/WCP2442. Kew Observatory began life as King’s Observatory (completed for George II to observe the 1769 Transit of Venus, 3 June 1769) and was located in Richmond. In 1842 the (by then empty) building was taken over by the British Association for the Advancement of Science and became known as the Kew Observatory; eventually passing to the Royal Society in 1871. See Scott (1885).

25 Wallace to Galton, 7 February 1891. “WCP4137,” in Beccaloni, G. W. ed., *Ɛpsilon: The Alfred Russel Wallace Collection* accessed on 7 October 2024, https://epsilon.ac.uk/view/wallace/letters/WCP4137. In a similar vein see Wallace to Galton, 13 February 1891. “WCP4138,” in Beccaloni, G. W. ed. *Ɛpsilon: The Alfred Russel Wallace Collection* accessed on 7 October 2024, https://epsilon.ac.uk/view/wallace/letters/WCP4138.

26 The Evolution Committee was established in 1894 as the Committee for Conducting Statistical Inquiries into the Measurable Characteristics of Plants and Animals, with Galton as Chairman. From 1897 it was known as the Evolution Committee (Plants and Animals). See GALTON 2/5/4, Evolution Committee (Plants and Animals) of the Royal Society, 1890–1897, Special Collections, UCL. On the Eugenics Laboratory see also Farrall (2019).

27 Wallace to Galton, 3 February 1891. “WCP1434,” in Beccaloni, G. W. ed., *Ɛpsilon: The Alfred Russel Wallace Collection* accessed on 7 October 2024, https://epsilon.ac.uk/view/wallace/letters/WCP1434.

28 Galton to Wallace, 12 Feb. 1891. “WCP2442,” in Beccaloni, G. W. ed. *Ɛpsilon: The Alfred Russel Wallace Collection* accessed on 7 October 2024, https://epsilon.ac.uk/view/wallace/letters/WCP2442.

29 Wallace to Galton, 3 February 1891. “WCP1434,” in Beccaloni, G. W. ed. *Ɛpsilon: The Alfred Russel Wallace Collection* accessed on 7 October 2024, https://epsilon.ac.uk/view/wallace/letters/WCP1434. Four years later we find him regretting that Darwin had not “left money for such experiments instead of for that huge Kew Catalogue of Plants which is being made so expensive and bulky that it will be almost useless for the purpose he intended it for & really money wasted.” Wallace to Galton, 6 February 1895. “WCP4136,” in Beccaloni, G. W. ed. *Ɛpsilon: The Alfred Russel Wallace Collection* accessed on 7 October 2024, https://epsilon.ac.uk/view/wallace/letters/WCP4136.

30 Wallace to Galton 2 December 1896. “WCP4134,” in Beccaloni, G. W. ed. *Ɛpsilon: The Alfred Russel Wallace Collection* accessed on 7 October 2024, https://epsilon.ac.uk/view/wallace/letters/WCP4134. See also Wallace to Edward Bagnall Poulton 2 December 1896. “WCP4406,” in Beccaloni, G. W. ed. *Ɛpsilon: The Alfred Russel Wallace Collection* accessed on 7 October 2024, https://epsilon.ac.uk/view/wallace/letters/WCP4406.

31 Wallace to Galton 1 Dec. 1893. “WCP4141,” in Beccaloni, G. W. ed. *Ɛpsilon: The Alfred Russel Wallace Collection* accessed on 7 October 2024, https://epsilon.ac.uk/view/wallace/letters/WCP4141.

32 Wallace was not averse to making statistical arguments. He used statistics in his biogeography and was an early proponent of the use of statistics in epidemiology, where he deployed them to some effect in making his case against vaccination. See Wallace (1889b).

33 In 1891 Wallace had tried to flatter Galton by telling him: “I am not an experimenter as you are.”. Wallace to Galton 7 Feb. 1891. “WCP4137,” in Beccaloni, G. W. ed. *Ɛpsilon: The Alfred Russel Wallace Collection* accessed on 7 October 2024, https://epsilon.ac.uk/view/wallace/letters/WCP4137.

34 *Inquiries* consisted of almost forty short papers written in the years after the publication of *Hereditary Genius* (1869). Together the papers, which range widely in subject matter, constituted a working out of Galton’s position. It is thus appropriate that this was the publication in which he first coined the term eugenics to encompass his work, and that the term was first used in a section concerned with “Bodily Qualities.” Galton (1883 p. 17 and p. 17n). On the anthropometric laboratory see Lundgren (2013).

35 Galton to Wallace, 24 May 1890. “WCP2755,” in Beccaloni, G. W. ed. *Ɛpsilon: The Alfred Russel Wallace Collection* accessed on 7 October 2024, https://epsilon.ac.uk/view/wallace/letters/WCP2755.

36 Galton to Wallace, 24 May 1890. “WCP2755,” in Beccaloni, G. W. ed. *Ɛpsilon: The Alfred Russel Wallace Collection* accessed on 7 October 2024, https://epsilon.ac.uk/view/wallace/letters/WCP2755.

37 Wallace to Galton, 6 February 1895. “WCP4136,” in Beccaloni, G. W. ed. *Ɛpsilon: The Alfred Russel Wallace Collection* accessed on 7 October 2024, https://epsilon.ac.uk/view/wallace/letters/WCP4136.

38 There was an uncharacteristic element of personal dislike in his response to Bateson. When the two had first met in Cambridge in spring 1894 Wallace was left cold by the younger man’s “cock-sure” arrogance, “cool bumptiousness,” and “high opinion of himself.” Wallace to Poulton 8 September 1894. “WCP4397,” in Beccaloni, G. W. ed. *Ɛpsilon: The Alfred Russel Wallace Collection* accessed on 7 October 2024, https://epsilon.ac.uk/view/wallace/letters/WCP4397. This fits the impression often created by Bateson’s “large, imposing, frame mixed with his overbearing conservative and/or romantic idealism and penchant for disdainful treatment of adversaries.” See Peterson (2008, p. 268).

39 Wallace to Poulton, 8 September 1894. “WCP4397,” in Beccaloni, G. W. ed. *Ɛpsilon: The Alfred Russel Wallace Collection* accessed on 7 October 2024, https://epsilon.ac.uk/view/wallace/letters/WCP4397. See Gilham (2001b).

40 Wallace similarly opposed Weldon’s claim that “natural selection is in most cases an imperfect agent,’ by arguing that whilst short term studies could produce “the appearance of imperfect adjustments,” longer-term investigations would demonstrate a more significant “underlying permanent adjustment,” Weldon’s misunderstanding, said Wallace, arose from a failure to appreciate that natural selection constantly varied the “locus of variation:” it did not, that is, operate at a constant rate or with the same severity at all times (Wallace 1894b).

41 Wallace to Galton, n.d. 1895. “WCP4144,” in Beccaloni, G. W. ed. *Ɛpsilon: The Alfred Russel Wallace Collection* accessed on 7 October 2024, https://epsilon.ac.uk/view/wallace/letters/WCP4144.

42 Wallace to Poulton, 8 Sept 1894. “WCP4397,” in Beccaloni, G. W. ed. *Ɛpsilon: The Alfred Russel Wallace Collection* accessed on 7 October 2024, https://epsilon.ac.uk/view/wallace/letters/WCP4397.

43 Wallace to Raphael Meldola, 31 Dec 1895. “WCP4531,” in Beccaloni, G. W. ed. *Ɛpsilon: The Alfred Russel Wallace Collection* accessed on 7 October 2024, https://epsilon.ac.uk/view/wallace/letters/WCP4531. Wallace had made the same point almost thirty years earlier: “Fully to grasp what is involved in that question demands a knowledge of details, which it requires years of study to amass; and without such knowledge the acutest and most comprehensive intellect will not suffice to solve so intricate a problem” (Wallace 1867, p. 488). This is consistent with Jane R. Camerini’s judgment that Wallace’s “fieldwork functioned as a transformative process, a critical ‘passage’ that permitted him to become a knowledge-maker in natural science” (Camerini 1996, p.44).

44 Although Wallace did not always agree entirely with Darwin. See, for example, Wallace (1889a p. 125).

45 Not everyone was convinced; Huxley remained a saltationist. See his review of the *Origin* in which he complained that Darwin underestimated the evolutionary importance of “sports” – “Nature does make jumps now and then, and a recognition of the fact is of no small importance in disposing of many minor objections to the doctrine of transmutation.” See Huxley (1860, p. 569). See also Lyons (1995).

46 DCP-LETT-5648 Darwin to Wallace 12 & 13 October 1867.

47 Prior to publication, Wallace and Darwin had a brief misunderstanding about the relative importance of “‘individual differences” and “single variations.” See DCP-LETT 6567 Darwin to Wallace 22 January 1869 and DCP-LETT-6583 Wallace to Darwin, 30 January 1869. The exchange ended with Darwin apologizing for having “expressed myself atrociously” and telling Wallace “I like much to be in accord with you.” Darwin’s explanation, moreover, confirms his distance from what would constitute a eugenic approach: “I believe I was mainly deceived by single variations offering such simple illustrations, as when man selects.” DCP-LETT-6591 Darwin to Wallace, 2 February 1869.

48 Wallace “protest[ed], *very strongly*” against the suggestion that he diverged from Darwin. Although he admitted that as more became known about the “extreme rigidity” of natural selection it was possible “to go one step farther than Darwin was able to do, and to doubt whether ‘single variations’ *ever* originated a natural ‘species’” (Wallace 1908b, pp. 716–717).

49 On Darwin and birth control see Peart and Levy (2008).

50 DCP-LETT-7154 Darwin to Wallace, 31 March 1870.

51 Wallace’s paper provided the jumping-off point for Greg 1868; although key aspects of Greg’s argument had been anticipated in Clemence Royer’s introduction to her 1862 French translation of Darwin’s *Origin*. See Harvey (1997).

52 Wallace described “Human Selection” as “the first scientific application” of the ideas he derived from Bellamy. Wallace 1905, p. 266.

53 The interview elicited a furious response from the eugenicist S. A. K. Strahan, author of *Marriage and Disease* (1892). See Strahan (1894) and Wallace’s reply (Wallace 1894a).

54 DCP-LETT-5140 A. R. Wallace to C. Darwin, 2 July 1866.
